# Immune responses underpinning acute co-infections with unrelated viruses: timing and location matter

**DOI:** 10.1093/intimm/dxaf018

**Published:** 2025-03-24

**Authors:** Isabelle Jia Hui Foo, Lukasz Kedzierski, Katherine Kedzierska

**Affiliations:** Department of Microbiology and Immunology, The Peter Doherty Institute for Infection and Immunity, 792 Elizabeth St, Melbourne VIC 3000, The University of Melbourne, Melbourne, Victoria, Australia; Department of Microbiology and Immunology, The Peter Doherty Institute for Infection and Immunity, 792 Elizabeth St, Melbourne VIC 3000, The University of Melbourne, Melbourne, Victoria, Australia; Department of Microbiology and Immunology, The Peter Doherty Institute for Infection and Immunity, 792 Elizabeth St, Melbourne VIC 3000, The University of Melbourne, Melbourne, Victoria, Australia

**Keywords:** concurrent infection, immunology, virus-virus interaction

## Abstract

Immunity to viral infections is generally studied in isolation by measuring immune responses towards a single virus. However, concurrent or sequential viral co-infections can occur in a single host. Viral co-infections can impact anti-viral immunity by altering protective responses and driving immunopathology. Understanding immune mechanisms towards co-infections with unrelated viruses is highly relevant to treatment and prevention. There is, however, a paucity of data on immune responses towards viral co-infections, especially with unrelated viruses. Most commonly studied viral co-infections include chronic viruses, such as hepatitis B, hepatitis C, and human immunodeficiency virus, as well as viruses infecting the same tissues, including respiratory viral co-infections. However, the immunological consequences of co-infections with unrelated acute viruses are less understood, especially for viruses affecting different anatomical sites. As co-infecting viruses can have a more pronounced impact on human health compared to infection with a single virus, understanding immune responses and, especially, the impact of timing, sequence, and location of viral co-infections is of key importance. This review provides an overview of the current knowledge on acute viral co-infections with unrelated viruses, underpinning immune mechanisms, and implications for vaccination regimens.

## Teaser

How virus co-infections affect immune responses

## Graphical Abstract

**Graphical Abstract:**
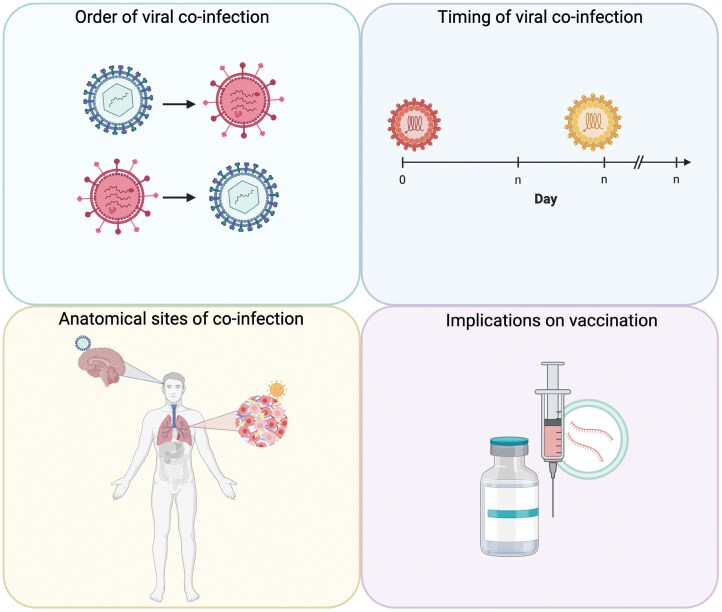

## Introduction

Infections with more than one pathogen (bacteria, virus, fungus, parasite, and protozoa) in a single host are defined as co-infections. Co-infections of global medical importance have been well documented in individuals infected with human immunodeficiency virus (HIV), tuberculosis, hepatitis viruses, dengue virus (DENV), malaria, and *Leishmania*. Similarly, concurrent or sequential infections with respiratory pathogens, including influenza A viruses (IAVs), rhinovirus (RV), and human coronaviruses, can occur in a single host because of the global co-circulation of the respiratory viruses, especially during winter epidemic months (1–3). Co-infections can also spread via insect vectors (e.g. mosquitoes) carrying multiple pathogens (4–6). Furthermore, contaminated blood products and used hypodermic needles can be a source of co-infections. A total of 90% of intravenous drug users living with HIV are also infected with the hepatitis C virus (HCV) (7). Co-infections with pathogens transmitted via different modes, e.g. respiratory and insect-borne pathogens, is increasingly common in areas with high burdens of both diseases (6, 8–11).

Immunity to infections is generally studied in isolation by measuring immune responses towards a single pathogen. As viral co-infections can impact anti-viral immunity by altering protective responses and driving immunopathology, understanding immune mechanisms towards co-infections is key to the treatment and prevention of infectious diseases. There is, however, a paucity of data on immune responses towards viral co-infections, especially with unrelated viruses. The universal burden of acute viral co-infection on human health is still unknown. This review focuses on co-infections with acute unrelated viruses, describes virological outcomes, and immunological consequences of acute viral co-infections, as well as factors that determine protective immunity versus immunopathology.

## Viral interference, enhancement, and accommodation

Viruses are the most common biological entities globally (12, 13). Thus, it is not surprising that infections with more than one virus, viral strain or subtype at the same time, or sequentially, occur in a single host, leading to viral co-infections (14). The capacity of two or more viruses to infect a single host depends on a number of factors, including host immune responses, which is the topic of the current review (15).

When a single cell is co-infected with different viruses, viral interference is the most common outcome. This occurs when one virus competitively suppresses other co-infecting viruses by affecting viral infection/replication, and as a result, the cell becomes resistant to the second superinfecting virus (16). The most common form of innate viral interference is mediated by interferons (IFNs). IFN activation leads to the induction of IFN-stimulated genes, resulting in the activation of anti-viral pathways. Together, they regulate the activity of innate immune mediators that can non-specifically block virus replication. Non-IFN-mediated viral interference is a virus-induced cellular state of resistance to subsequent viral infection, which can happen between related or unrelated viruses. In this case, two viruses compete for metabolites, replication sites, or other host factors that support viral replication.

Viral interference usually occurs at a specific stage of the viral replication cycle such as attachment, entry, genome replication, and budding. This was demonstrated by Muturi and Bara showing that sequential co-infection with DENV and Sindbis virus (SINV) in invertebrate *Aedes albopictus* (C6/36) cell line led to reduced DENV replication. However, DENV had antagonistic effects on SINV replication in a simultaneous co-infection setting, demonstrating that both viruses interfere with the replication of the other (17). Similarly, the effect of viral interference was also observed *in vivo*, where co-infection of domestic ducks with virulent Newcastle disease virus (vNDV) and low pathogenicity avian influenza virus decreased shedding of vNDV, despite no changes in clinical symptoms between single infection and co-infection. Co-infection also reduced transmission of vNDV to uninfected ducks housed with the inoculated ducks (18). Finally, simultaneous co-infection with LaCrosse virus (LACV) and SINV had an antagonistic effect on both viruses in baby hamster kidney cells, whereby viral interference occurred at the replication stage for both viruses (19).

Conversely, viral co-infections can also lead to enhanced virus replication. This has been demonstrated by co-infection of C6/36 mosquito cells with LACV and SINV, which led to enhanced SINV replication (19). Additionally, Goto *et al*. (20) showed that initial infection with human parainfluenza virus 2 facilitated IAV replication and modulated pathological outcomes in Vero cells, but not vice versa, demonstrating that the order of viral infection affects disease outcome. Contrary to enhanced virus replication or elimination of virus, infected cells can harbour virus for prolonged periods of time, thus transmitting virus to a new susceptible host or having no effect on the replication of the respective viruses, known as viral accommodation (21). Previous studies demonstrated that persisting viruses isolated from mammalian or mosquito cell lines can disrupt the growth of the wild-type counterparts (22–25). Therefore, persisting viruses have a reduced capacity to kill virus-infected cells because of their inability to shut down host machinery. Co-infection with multiple viruses in arthropods carrying DENV and densovirus at the same time is an example of an active persistent infection (viral accommodation), with no signs of adverse outcome (26). However, the molecular mechanisms underlying viral accommodation remain elusive.

## Acute viral co-infections

A number of studies have previously explored interactions between chronic viral pathogens. For instance, co-infections involving hepatitis B virus and HCV have been frequently studied due to their significant impact on liver disease progression and treatment outcomes (27–30). Similarly, the co-occurrence of other chronic viruses, such as cytomegalovirus, HPV, or viral hepatitis in people living with HIV is well-documented, revealing complex interactions that can affect disease severity and patient management (7, 31–33).

On the other hand, the phenomenon of acute viral co-infection remains relatively unexplored. Although acute viral infections with, for example, influenza viruses or respiratory syncytial virus, are well-studied individually, their co-infection with other acute viruses, especially those that infect different and unrelated organs and anatomical sites, has not been thoroughly investigated. This gap in knowledge hampers our understanding of how co-infections might alter disease progression, impact immune responses, or affect treatment outcomes. Here, we discuss reports and findings of acute viral co-infections in humans and animal models, affecting either the same or different organs.

### Co-infecting viruses targeting the same anatomical sites

The majority of acute viral co-infections occur via the same route and/or affect the same organs. A study conducted during the 2009 H1N1 IAV pandemic showed that 13% of hospitalized patients were co-infected with another respiratory virus. Patients co-infected with IAV and seasonal human coronavirus had exacerbated respiratory disease compared to patients infected with IAV only (3). Conversely, patients co-infected with IAV and RV had lower disease severity despite having comparable IAV titres across single- and co-infection groups (3). Co-infections of the respiratory tract with measles and influenza virus were reported in Africa, with patients developing complications such as pneumonia and enteritis (34).

Co-circulation of multiple subtypes and sub-lineages of influenza viruses can lead to concurrent infections of IAV and influenza B virus (IBV), which were detected in surveillance programs (35–37). While the occurrence is rare, concurrent infection of influenza A and B makes up 1.6% (10 out of 609 patients) detection in surveillance, and co-infection in this case was not associated with severe disease outcome, underlying conditions, or vaccination status (35). Similarly, another case report found 0.6% (1 out of 159 patients) detection of influenza A and B co-infection (37). CD8^+^ T cells cross-reactivity across IAV, IBV, and influenza C that recognize the conserved PB1_413_ epitope could explain why IAV and IBV co-infection may not be severe (38).

The emergence of severe acute respiratory syndrome coronavirus 2 (SARS-CoV2) that causes coronavirus disease (COVID-19) in recent years has also led to increased co-infections with multiple respiratory viruses, especially influenza viruses (39–42). In addition, co-infections of arthropod-borne viruses (arboviruses) which generally have broad tissue tropism (43), are common due to the co-distribution of these viruses and the ability of vectors (e.g. mosquitoes) to carry more than one virus. DENV is one of the most medically significant flaviviruses to date (44), with *Aedes* mosquitoes serving as its primary vector. Additionally, other arboviruses such as chikungunya (CHIKV) and Zika (ZIKV) are endemic to regions, where DENV is also present and often share the same vectors. However, the burden and prevalence of arboviral co-infections remain underreported because symptoms presented by infected patients are similar. Although there are many reports of DENV-CHIKV and DENV-ZIKV co-infections in humans (45–47), the disease outcomes of these co-infections remain unclear. However, a study involving experimentally co-infected rhesus macaques with ZIKV and DENV-2 found that both viruses replicated without exacerbating the symptoms of either infection (48).

### Co-infecting viruses targeting distinct anatomical sites

Conversely, acute viral co-infections that affect different organs or primary infection sites often involve distinct routes of transmission and are frequently underreported due to overlapping or milder clinical symptoms compared to the other co-infecting virus. A sudden reemergence of the monkeypox virus (MPV) global outbreak in 2022 has recently resulted in co-infections with SARS-CoV2 in patients infected with MPV (49). MPV spreads through close contact with an infected individual, contaminated fomites, or with infected animals, whereas SARS-CoV2 is transmitted through aerosol droplets (50, 51). Meta-analysis by El-Qushayri *et al*. (49) suggests that while all three cases of co-infection were hospitalized, none experienced severe outcomes, and all were discharged after a full recovery. These results indicate that SARS-CoV2 and MPV co-infection does not result in more severe outcomes, even though all the patients in this study had multiple co-morbidities, which are known to be significant risk factors for severe COVID-19 complications (49, 52).

As mentioned above, co-infections with respiratory viral co-infections (1, 2, 53) or arbovirus-arbovirus co-infections (45–47, 54, 55) are relatively well documented and studied. However, limited data exist on how co-infections with respiratory viruses and arboviruses affect underlying immune responses and thereby disease outcomes. Most of our knowledge to date encompasses influenza and DENV co-infections. Seasonal IAV and DENV epidemics regularly overlap in tropical and subtropical regions such as Brazil (56, 57). Throughout the 2009 H1N1 pandemic, influenza-DENV co-infections were reported and identified as a risk factor for severe disease. Furthermore, arboviral co-infection could increase the neurotropism of respiratory pathogens such as IAV by affecting the central nervous system (CNS) immunity (58). It was also discovered that non-neurotropic and neurotropic influenza viral strains could enter the brain in the absence of co-infection, inducing neuroinflammation and compromising the BBB (59). Since the COVID-19 pandemic, SARS-CoV2 and DENV co-infection have been on the rise (11, 60, 61), with co-infected patients experiencing moderate to severe respiratory disease compared to those infected only with SARS-CoV2. Co-infected patients were reported to need mechanical ventilation as well as ICU admission and hospitalization (61).

**Figure 1.**
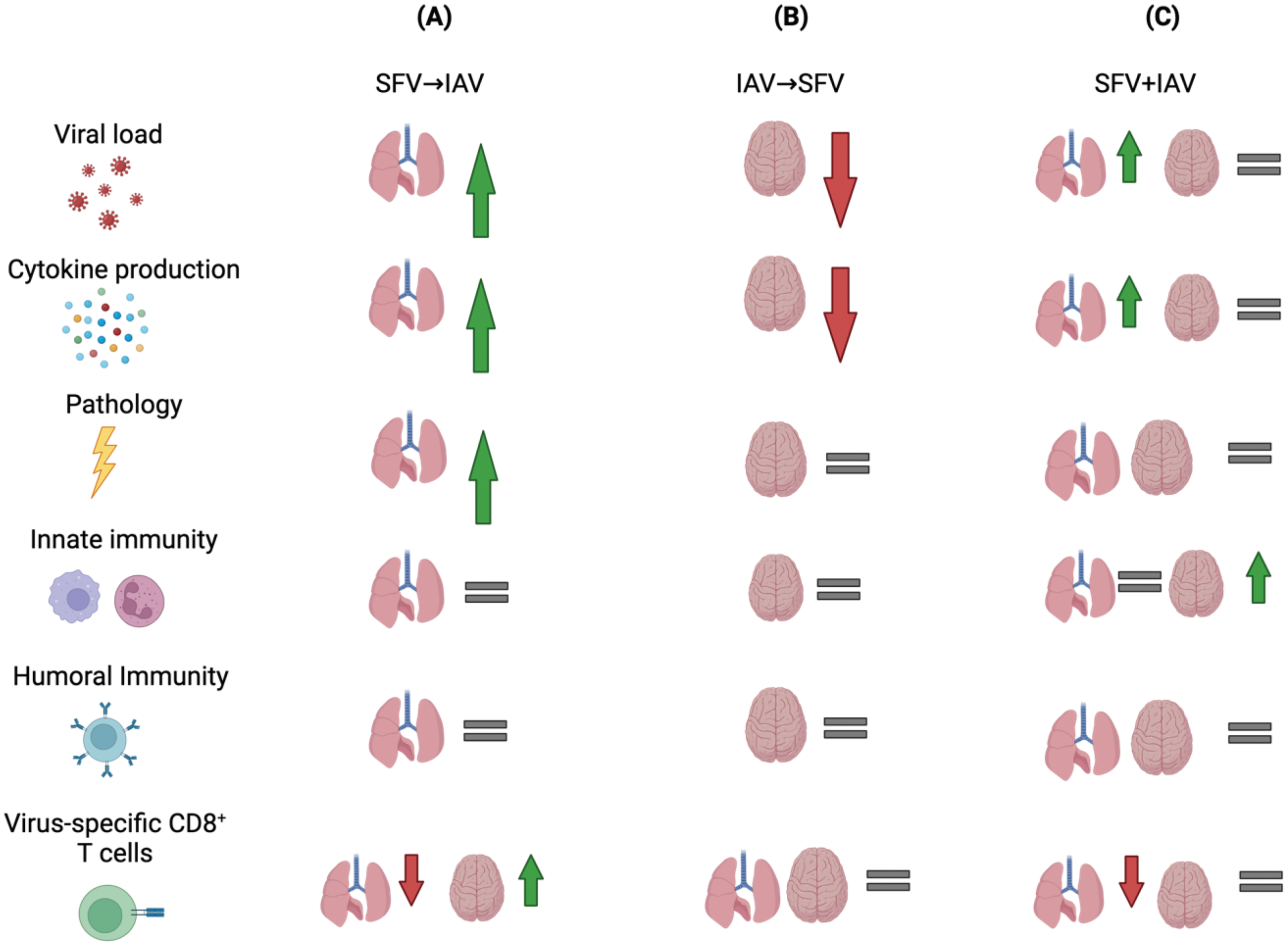
An example of co-infection studies between two acute, unrelated viruses, Semliki Forest virus (SFV) and influenza A virus (IAV) affecting two major organs: lungs and brain. Outcomes of co-infections are different across different measurements of immune parameters (rows) and depend on the order and timing of infection (columns). Viruses were given sequentially (A) SFV followed by IAV, (B) IAV followed by SFV, or (C) both viruses simultaneously. Upward arrows indicate an increase; downward arrows indicate a decrease; equal signs indicate no change.

While DENV is not typically neurotropic, its geographical distribution overlaps with other neurotropic arboviruses, hence the likelihood of influenza-arbovirus co-infections occurring frequently is relatively high. Given that the clinical manifestations of encephalitic arboviruses can be mild, asymptomatic, or overlap with those of respiratory viruses, it is unsurprising that there are very few reports on co-infections between respiratory viruses and encephalitic arboviruses. Our recent study in mice demonstrated that co-infections with the encephalitic arbovirus Semliki Forest virus (SFV) and IAV resulted in more severe disease outcomes compared to single infections (Fig. 1) (62), highlighting the importance of investigating immune responses underpinning co-infections with acute unrelated viruses infecting distinct anatomical sites, impacting disease severity and progression.

## Timing and sequence of co-infections impact anti-viral immunity and immunopathology

Pre-existing immunity to previously encountered pathogens can modify responses against other novel pathogens, termed as “heterologous immunity” and can happen between related or unrelated viruses (63, 64). The heterologous immune response can result in protective immunity or, conversely, can lead to immunopathology (65–68). Optimal immune responses are pivotal for effective pathogen control in heterologous infections. However, viral co-infections can alter memory T cell responses leading to decreased protection and enhanced immunopathology upon re-infection (69). Here, we discuss how the timing and sequence of co-infection affect anti-viral immunity.

### Sequence of co-infections affects immune responses and disease severity

Prior exposure to IAV has been shown to alleviate the disease severity of RV infection (70). A viral co-infection experiment in a mouse model demonstrated that RV infection, followed by a medium dose of IAV-PR8 infection 2 days later, delayed and reduced disease severity when compared to mice that were given RV and IAV-PR8 at the same time. Conversely, when mice were given RV 2 days after receiving IAV-PR8, viral co-infection exacerbated IAV-PR8 disease (71). Similarly, in a co-infection study where both viruses target different organs, the authors demonstrated that preceding SFV infection negatively impacted immune responses to the subsequent influenza virus infection (Fig. 1A). Immune responses to IAV were markedly perturbed, leading to altered T cell trafficking, with IAV-specific CD8^+^ T cells found in the brain in SFV→IAV co-infected mice coupled with reduced magnitude of these IAV-specific CD8^+^ T cells in the lungs. SFV→IAV sequential co-infection also resulted in elevated inflammation in the lungs, increased viral load, as well as aggravated lung pathology. This was associated with impaired lung IAV-specific CD8^+^ T cell responses, stemming from suboptimal CD8^+^ T cell activation and proliferation in draining lymph nodes, as well as antigen-presenting cell (APC) paralysis (Fig. 1A) (62).

Conversely, previous IAV infection alleviated arbovirus encephalitis in mice (Fig. 1B). IAV infection preceding SFV infection attenuated the subsequent SFV disease by systemic induction of type I IFN, resulting in reduced infectious SFV titres and decreased levels of cytokines and chemokines in the CNS (72), which contrasts to the reversed order of infections (Fig. 1B). Schmid *et al*. (73) demonstrated that co-infection with influenza virus followed by DENV 2 days later impairs host immune responses, thus failing to control DENV titres, leading to severe lung damage in mice. This was mediated by impaired host mRNA responses in the lung and reduced recruitment of monocytes, which facilitated pathogenesis. However, infecting mice with DENV followed by IAV (DENV→H1N1) did not result in as a severe disease as H1N1→DENV (73). Collectively, these studies highlighted that the sequence of viral co-infections can greatly impact the magnitude and nature of immune responses and hence disease outcomes.

### Timing of viral co-infections

Simultaneous co-infection with Ectromelia virus (ECTV), otherwise known as mousepox virus, and lymphocytic choriomeningitis virus (LCMV) in mice led to decreased ECTV viral load and ameliorated ECTV-induced disease compared to the single infections of both immunizing viruses (74). It is postulated that this protection is mediated by the induction of type I IFN by LCMV, overwhelming ECTV mechanisms for type I IFN suppression. However, this resulted in diminished LCMV-specific CD8^+^ T cell responses and the formation of a TNF-deficient effector-memory phenotype that was less protective (75, 76). Previous studies involving influenza and murine herpes virus reported that co-infection resulted in alleviated respiratory symptoms due to influenza virus infection (77).

Another co-infection study in mice demonstrated that simultaneous infection with acute LCMV and Pichinde virus (PICV) (LCMV + PICV) resulted in reduced protective immunity and increased immunopathology compared to single-virus infections when the mice were re-challenged with their respective immunizing viruses (LCMV + PICV infection, LCMV challenge and LCMV + PICV infection, PICV challenge) (69). The authors demonstrated that PICV immune response dominated in LCMV + PICV co-infections, resulting in lower LCMV-specific CD8^+^ T cells which subsequently led to delayed LCMV viral clearance following rechallenge with chronic LCMV. Similarly, immunopathology was observed in LCMV + PICV co-infected mice upon PICV re-exposure because of non-protective cross-reactive memory CD8^+^ T cells (69).

The simultaneous exposure to peptides from two different viruses creates a competitive environment for APCs to present antigens of each virus. Fewer APCs would lead to increased competition among T cells for access to APC binding sites, resulting in qualitative and quantitative changes in immune responses and immunopathology (69, 78). Studies demonstrated that sequential SARS-CoV2 and IAV co-infections, and vice versa, increase disease severity in mice (40, 42). This can be exemplified by the study by Achdout *et al*. (42) that found that prior infection with PR8 IAV followed by SARS-CoV2 2 days later was lethal in mice. However, increasing the time between secondary SARS-CoV2 alleviated disease severity and thus mortality (42). Finally, our study demonstrated that simultaneous co-infection with SFV + IAV did not lead to exacerbated or attenuated disease compared to the single viral infection control groups (Fig. 1C) (79).

We found that inflammation and overall immune responses in the brain in simultaneous co-infection were comparable to that of SFV-only, potentially due to similar SFV titres across both infection types. In comparison, despite enhanced IAV replication in the lungs in SFV + IAV infection, we did not observe exacerbated lung pathology in SFV + IAV compared to IAV-only infection. However, the magnitude of IAV-specific CD8^+^ T cell responses in the lungs was lower compared to that of IAV-only infection (Fig. 1C). These results of higher lung IAV viral titre and lower number of IAV-specific CD8^+^ T cells in the lungs of simultaneously co-infected mice mirror what was observed in our previous co-infection study (62), with the exception of aggravated lung pathology observed in sequential SFV→IAV co-infection (Fig. 1A and C). Thus, we concluded that the timing of viral co-infection is pivotal in determining immune responses and disease outcomes, demonstrated by our finding of simultaneous infection of SFV and IAV without greater lung pathology (79).

Altogether, these studies provide evidence that the delicate balance between protective anti-viral immunity versus immunopathology is dictated by the timing and sequence of viral co-infections as well as heterologous immunity, viral epitopes, and host factors. Given the intricate interplay between viral infections and the immune system, understanding immunity to viral co-infections remains a crucial area of research for developing effective treatments and vaccines.

### Implications on vaccinations

Similar to viral co-infections, vaccination efficacy relies on factors like age, pre-existing immunity, stages of immunological maturation, mode of delivery, and vaccination schedule that may determine interference (80). A Nigerian study reported that children who received smallpox, measles, and yellow fever vaccines simultaneously at separate sites responded adequately to all three vaccines. However, when these same immunized children were given an additional diphtheria–pertussis–tetanus vaccine, there was a decrease in measles seroconversion rates (81), suggesting vaccine interference. Similarly, a randomized study conducted by Nascimento Silva and colleagues demonstrated that the order of vaccination matters. They found subjects with concomitant administration of yellow fever vaccine (YFV) and measles–mumps–rubella (MMR) vaccine at different sites of injection mounted a weaker immune response to all pathogens demonstrated by the lower IgG antibody seroconversion rates (90% for rubella, 70% for yellow fever, and 61% for mumps). In comparison, subjects who received MMR vaccine followed by YFV 30 days later had a greater IgG seroconversion rate (97% for rubella, 87% for yellow fever, and 71% for mumps) (82).

Like many co-infections that may not lead to adverse outcomes, vaccination studies did not find evidence of interference (81, 83–86). However, that is not to say that the immunological mechanisms of co-infections or vaccinations are well elucidated. Some vaccines today still require seasonal updates to accommodate and combat the highly evolving viruses. Future vaccine strategies may consider administrating vaccines of different viruses on different arms, as it was observed that administration of both influenza and SARS-CoV2 vaccines at the same site (arm) led to a small detrimental effect on the fold-increase in SARS-CoV2 antibody levels (87). Otherwise, a universal vaccine, either by the mosaic vaccine (88), antigen multimerization (89, 90), or antigen coupling (91) of multiple viruses may be considered for effective protection against diverse viruses. Because of the complex interaction of our environment and immune system, immunity towards viral co-infections should remain a research priority.

## Conclusion

Collectively, the studies reviewed here suggest that viral co-infections have significant biological and epidemiological consequences. This is especially relevant for individuals from higher-risk groups, including children, elderly, pregnant women, individuals with co-morbidities, and First Nations populations, being more at risk of severe virus infections (70, 92–97). Therefore, understanding the effects of co-infections is an emerging and exciting field in immunology and virology. The knowledge gained from such studies will be relevant and of vital importance to improve diagnostics, vaccines, and anti-viral therapies.
